# Preventing Alcohol Abuse Through Social Networking Sites: A First Assessment of a Two-Year Ecological Approach

**DOI:** 10.2196/jmir.4233

**Published:** 2015-12-10

**Authors:** Valentin Flaudias, Ingrid de Chazeron, Oulmann Zerhouni, Jordane Boudesseul, Laurent Begue, Renaud Bouthier, Christel Lévrier, Pierre Michel Llorca, Georges Brousse

**Affiliations:** ^1^ CHU Clermont-Ferrand Pôle Psychiatrie B Clermont-Ferrand France; ^2^ Clermont Université EA NPsy-Sydo Université d'Auvergne Clermont-Ferrand France; ^3^ LIPPC2S Université Grenoble Alpes, F-38000 Grenoble France; ^4^ Association Avenir Santé Lyon France

**Keywords:** social networking, primary prevention, alcohol consumption, students

## Abstract

**Background:**

Prevention strategies to reduce alcohol use/consumption among young people are crucial to reducing alcohol-related deaths and preventing disease. This paper focuses on the effectiveness of a social networking site (SNS) alcohol prevention program targeted toward young people.

**Objective:**

We hypothesized that the program would diminish the relation made by participants between alcohol and festive moments, and would result in a reduction of their declared consumption of alcohol at festive moments during the program. We also explored the interaction with the prevention program that was the most efficient.

**Methods:**

The prevention program took the form of 3 lotteries over 2 years. The participants periodically received prevention messages, particularly on alcohol and festive moments (eg, videos on Facebook and short message service [SMS] text messages on their mobile phones). For the 3 periods, the participants had to answer questions exploring the level of their belief that alcohol consumption and festive moments are highly associated. A control group that did not participate in the prevention program was asked the same questions over the same number of days for the first 2 periods. During the second period, the participants were asked to answer questions about their alcohol consumption during parties. During the third period, we explored the interaction with the prevention program on the reduction of their belief that alcohol consumption and festive moments are associated.

**Results:**

A total of 651 participants (age: mean 22.24, SD 4.10 years; women: n=430) during the first period, 301 participants (age: mean 21.27, SD 3.07 years; women n=199) during the second period, and 305 (age: mean 22.41, SD 4.65 years; women: n=190) during the third period correctly completed the survey. For the control group, 69 students completed the survey during the first period (age: mean 18.93, SD 1.14 years; women: n=59) and 50 during the second (age: mean 20.78, SD 1.94 years; women: n=45). We observed a significant reduction in the association of alcohol with festive moments in the participants over the 2 years (period 1: *z*=–4.80, *P*<.001; period 2: *z*=–2.11, *P*=.04; period 3: *z*=–2.30; *P*=.02), but not in the controls. We also observed a reduction in the number of glasses consumed during festive moments for the participants (*z*=–2.36, *P*=.02), but not for the controls during the second period. The third period showed that only the number of days since registration in the program had an impact on the reduction of the association of festive moments and alcohol consumption (*t*_21_=3.186, *P*=.005).

**Conclusions:**

The findings of this study suggest that the SNS prevention program is promising in preventing the association of alcohol with festive moments and, more generally, in impacting social norms.

## Introduction

Alcohol is the most harmful substance in the United Kingdom [1] and it is the third leading preventable cause of death in the United States [2]. Globally, approximately 76.3 million people have been diagnosed with alcohol use disorders. In most Western countries, alcohol use commonly begins during adolescence [3]. According to a French national survey (Enquête sur la Santé et les Consommations lors de l'Appel de Préparation À la Défense [ESCAPAD]), in 2011, 10% of 17-year-olds participated in regular drinking (at least 10 drinks during a month); 28% reported repeated drunkenness (having been drunk at least 3 times a year), 10.5% were drunk 10 or more times a year, and 53.2% reported having drunk more than 5 glasses of alcohol during one event within the last 30 days [4]. This survey shows an increase in binge drinking in this population. A recent review [5] defined *binge drinking* as “a pattern of drinking alcohol that brings blood alcohol concentration to 0.08 g/dL or above (≥5 for men or ≥4 for women in 2 hours) on more than one occasion within the past 6 months.” This massive consumption during a short period could have tragic short-term effects, such as accidents, violence, or ethyl coma [6]. Recent studies have also shown that this type of consumption seems to have a long-term impact on spatial working memory [7] and other neurocognitive functions [8]. In this paper, the focus is on festive moments, or event occasions. In their review, Mallett et al [9] showed that there are many events during student life. “Festive moments” include all these events: parties, celebrations, sport events, holidays, school breaks, and personally relevant events (see also [10]).

To better understand how the consumption of alcohol during festive moments is integrated, it is important to know how the representation of festive moments is communicated. The media has an important impact on young people about social norms regarding alcohol consumption [11], including that alcohol consumption is mandatory to enjoy a party [12].

Recently, the most important type of media for young people is the Internet, especially social network sites (SNSs). A total of 82% of French people younger than age 25 years use Facebook [13]. Facebook is currently the most popular SNS in the world, topping 1 billion active users with 580 million who engage with the site daily [13-15]. This platform represents an important strategic issue for communicating with young people.

One of the strengths of Facebook is that registered people become vectors of communication for messages posted on a page. Indeed, “liking” a page and commenting on the news will result in the commentary being displayed publicly so that their friends will, in turn, receive this information. Alcohol producers use various strategies on social networks to promote alcohol consumption (eg, interactive games, contests, videos sent to minors, Facebook pages). Nicholls [16] showed that social norms could be influenced by alcohol marketing strategies on SNSs and that “traditional notions of celebration play a key role.” Ridout et al [17] observed a normalization of alcohol consumption, particularly in the search for an acceptable social identity, because of the importance of the “drinker” status on SNS. Online interactions contribute to the normalization of youth consumption of alcohol [18]. A recent example regards a beer seller, who launched a lottery to win beer-related prizes. The participants had to register on their Facebook pages and then answer 3 questions about the company. These questions, of course, required the participant to explore the page or to search the Internet for answers. After having responded and participated in the lottery, this information was displayed on the Facebook pages of the participants, allowing their friends to access it as well. At the time this paper was written, this French page had 229,934 fans.

A recent study in the United States showed that teens who use SNSs at least once per day (70% of those aged 12-17 years use them for an average of 23 minutes per day) were 5 times more likely to use tobacco (10% vs 2%), 3 times more likely to consume alcohol (26% vs 9%), twice as likely to use marijuana (13% vs 7%), and almost 4 times as likely to be exposed to images of young people smoking, drinking, or using drugs [19].

Despite the success of these marketing strategies, few prevention programs have used SNSs. Capurro et al [15] recently reviewed 58 articles related to public health research on Internet sites. However, many of these studies focused on users and usage of SNSs rather than the effectiveness of this type of information on prevention.

The main objective of this paper was to evaluate a SNS-based prevention program [20] and show the results of its effectiveness in changing the representation of festive moments and, more particularly, in altering the high association of alcohol consumption and these moments. For this purpose, we thought that the participants in a 2-year existing SNS-based prevention program would show a weaker association between festive moments and alcohol consumption at the end of the program compared to the beginning. More importantly, we hypothesized that this difference would not be significant for a similar sample questioned at the same time, but who did not register for this program. Finally, our evaluation took into account the behavior (eg, activities) on the program’s SNS page to better understand the reduction in the association between alcohol and festive moments.

## Methods

### Description of the Prevention Program

This program aimed to reduce extreme alcohol consumption in a festive context among youth. To do this, we used 2 types of communication associated with festive contexts: the Internet through social networks (Facebook) and mobile phone services by sending short message service (SMS) text messages.

Concerning the Internet and, particularly, Facebook, the aim was to regularly spread prevention campaigns with a dedicated Facebook page entitled “Auvernight.” This page contained mostly videos, but also posters and slogans from national programs (eg, the program SAM for drivers, which was aimed toward choosing a sober driver in a group while they are partying) to challenge young people to change their festive habits. Over the 2-year period, approximately 43% (16/37) of these preventive messages directly concerned alcohol; others concerned drug consumption, road accidents, and sexually transmitted diseases. To make the page more attractive and representative of the festive world, we also posted a selection of local festive activities. To bolster interest in this page, we introduced a lottery each academic year (3 lotteries during the 2 years of the program) with attractive prizes (eg, a trip or a computer costing €3500 or approximately US $4000).

Because it is known that alcohol-related cognitions in long-term memory have a strong influence on drinking behavior (eg, expectancies toward alcohol [21,22]) and that alcohol-related contextual cues are likely to activate behavioral schemes associated with these expectancies [23], SMS text messages were sent as close in time as possible to key moments for festive events (eg, when approaching the weekend, at the beginning of school holidays) to ensure a maximal impact on drinking behavior while partying. The messages reminded the participants of a number of tips to reduce the risks potentially associated with alcohol and the negative effects of massive alcohol consumption. Of these SMS text messages, 71% (12/17) specifically concerned alcohol and 29% (5/17) were about other drug consumption. This approach was complementary to the approach used on the Internet in the sense that it was more situational (eg, sending a message during a festive moment such as a New Year celebration).

Regarding this type of communication, it is important to have a long-term perspective to increase the knowledge of this page in the target population. This is why a multiple year program was necessary. To maintain interest, 3 lotteries were organized during the 2 years of the program. Each lottery lasted 3 months. The first took place between February and June 2013, the second between April and June 2014, and the third between October and December 2014. The population could be different for each lottery, so we explored the results for each lottery period independently.

### Participants and Procedure

The participants in this program were recruited using emails sent to their personal mailboxes from a listing of the “Avenir Santé” association for their other prevention activities or by flyers distributed near the amphitheater or the library at the University of Clermont-Ferrand (France). This recruitment was conducted for each period. They had to complete an online registration. During this registration, participants answered questions to assess their views on alcohol consumption during festive moments. Three months later, they completed the questionnaires assessing the same questions. To be included in the lottery, they had to be registered on the Facebook page and they were informed that they would have to conserve the SMS text messages on their mobile phones to have an advantage because they would be asked questions about their content. This was done to ensure that they would read the SMS text messages. These participants composed the All Facebookers group.

In the first 2 periods, we chose to include a control group that was composed of students. For the first period, psychology undergraduates were recruited near the University of Clermont-Ferrand using the same methods and places as those used for the participants in the program. They completed the same questionnaire as the All Facebookers group at the same time. For the second period, psychology undergraduate students at the University Grenoble-Alpes (France), who were not registered in the lottery, were involved in the evaluation. They were recruited by email to complete the survey at the beginning and the end of the program. These students composed the control group. We had no control group for the third period.

To ensure that the control group was comparable with the All Facebookers group, we chose to pair students who participated in the prevention program (and so who were members of the All Facebookers group) by sex and age with students of the control group. This was the Paired Facebookers group.

Therefore, we had 3 groups: one group for all participants on Facebook (All Facebookers), students not registered for the prevention program (control), and paired participants/students (Paired Facebookers). The first period of this program took place between February and June 2013 (period 1), the second period between April and June 2014 (period 2), and the third period between October and December 2014 (period 3).

### Measurements

#### Period 1

After demographic data were collected (age and sex), the participants were asked, “On a scale from 0 to 10 (0=not at all, 10=absolutely), how much do you think that alcohol is necessary to have a successful party?” This question was asked online at the beginning and at the end of the program for the Facebookers and on paper for the control group.

#### Period 2

The 4 questions concerning party representations were “On a scale from 0 to 10 (0=not at all, 10=absolutely):

1. How much do you think that alcohol is necessary to have a successful party?
2. How much do you think that alcohol improves the mood of a party?
3. How much do you think that alcohol may lessen the interest of a party?
4. How much do you think a party is more successful without alcohol?”

To finish, we assessed their alcohol consumption for one party with the following question: “How many glasses do you drink when you are at a party?” The order in which the questions were presented was randomized for each participant. All participants and control group members answered online at the beginning and at the end of the program.

#### Period 3

The same questions as those asked during period 2 were used at the beginning and at the end of period 3. We added a memory question regarding the SMS text message. For this, we asked the participants to choose between a set of 10 propositions, of which 5 included correct content of the SMS text message that they had received. At the end of the program, the number of likes on the page was counted to measure the activity on the page.

### Statistical Analysis

The data were analyzed using SPSS version 19. The comparison between the pre- and posttests (at the beginning and at the end of the period) was computed with a nonparametric Wilcoxon test for the question associated with alcohol and festive moments, and for each sample (All Facebookers, Paired Facebookers, and control). Then, the difference between the pre- and posttests for this association in the Paired Facebookers and the control groups were compared with a Mann-Whitney nonparametric test. When significance was observed, a Cohen’s *d* was calculated to assess the effect size. The differences concerning the association of alcohol between these 2 populations at the beginning of the program were compared using a nonparametric Wilcoxon test. The same analysis was conducted for the number of glasses consumed at one festive moment for the pre- and posttests.

Concerning periods 2 and 3, Cronbach alpha analyses were completed to assess the reliability of all questions before a mean was calculated for the 4 questions regarding the association of alcohol with festive moments.

Finally, for the third period, a linear regression analysis was computed on the reduction of the association of alcohol with festive moments and the number of correct SMS text messages remembered, the number of days following registration in the program, and the number of Facebook likes as factors to better understand the reduction.

## Results

### Description of the Population and Reliability of Items

At the beginning of the first period, we had 866 participants. Three months later, at the end of the program, 651 had completed the questionnaires assessing the same questions (see Table 1). A total of 69 participants were in the control group after one was excluded because of missing data (ie, the questionnaire was not filled out completely); mean age was 18.93, SD 1.14 years and 59 (86%) were women. There were 651 in the All Facebookers group (age: mean 22.24, SD 4.10 years; women: n=430, 66.1%) and 69 in the Paired Facebookers group (age: mean 19.75, SD 0.94 years; women: n=59, 86%).

During the second period, 498 persons participated at the beginning of the program, 424 appropriately answered the 4 questions, and 301 of these correctly completed the 2 questionnaires at the beginning and at the end of the program (14.7%, 52/353 were excluded because they answered inconsistently on negative version questions). There were 21 participants common to the first period. Analyses were performed for the 2 populations: the 301 total participants and the 280 new participants. Thus, 301 participants composed the All Facebookers group (age: mean 21.27, SD 1.94 years; women: n=199, 66.1%) or 280 when we only observed new participants (age: mean 21.24, SD 3.12 years; women: n=185, 66.1%), 50 controls (age: mean 20.78, SD 1.94 years; women: n=45, 90%) and 50 Paired Facebookers (age: mean 20.80, SD 2.00 years; women: n=45, 90%).

In the third period, 452 persons participated at the beginning of the program and 305 appropriately answered the 4 questions and completed the 2 questionnaires at the beginning and at the end of the program (1 was excluded because he answered inconsistently on negative version questions). There were 25 participants who were also involved in the first or the second period. Analyses were performed on the 2 populations: the 305 total participants (age: mean 22.41, SD 4.65 years; women: n=190, 62.3%) and the 280 new participants (age: mean 22.30, SD 4.74 years; women: n=173, 61.8%).

Concerning the reliability of the items (see Table 2), all Cronbach alphas were greater than .70 for the second period and .80 for the third period, which could be considered “acceptable” [24].

### Association of Alcohol Consumption with Festive Moments

For the All Facebookers group, we observed a significant reduction in the association of festive moments with alcohol consumption between the beginning and the end of the program for the 3 periods of the evaluation (period 1: *z*=–4.80, *P*<.001; period 2: *z*=–2.11, *P*=.04; period 3: *z*=–2.3, *P*=.02) (see Table 1). Similar results were observed with only new participants for the second and third periods.

For the control participants, we observed that there was no significant difference between the association of alcohol with festive moments at the beginning and at the end of the program for the first and second periods (period 1: *z*=–0.35, *P*=.73; period 2: *z*=–0.73, *P*=.47).

Finally, when we compared the Paired Facebookers and the control groups concerning the differences between pre- and posttest results, we found a difference for the association of alcohol with festive moments only for the first period (*z*=–2.24, *P*=.02).

Concerning the pretest, we observed a difference between our control*s* and the Paired Facebookers for the first period (*z*=–2.13, *P*=.03), but not for the second (*z*=–0.33, *P*=.74).

**Table 1:** Comparison between pre- and posttest scores for association of alcohol and festive moments for each group during the 3 test periods and the mean number of glasses of alcohol consumed during one festive moment for periods 2 and 3.

Type of participants and characteristics of analyses		Period 1	Period 2				Period 3			
			All participants		New participants only		All participants		New participants only	
		Alcohol association	Alcohol association	Alcohol consumed during one festive moment	Alcohol association	Alcohol consumed during one festive moment	Alcohol association	Alcohol consumed during one festive moment	Alcohol association	Alcohol consumed during one festive moment
**All Facebookers**											
	n	651	301	301	280	280	305	305	280	280
	Pretest, mean (SD)	3.41 (2.72)	4.23 (2.29)	2.68 (1.64)	4.22 (2.26)	2.62 (1.62)	4.34 (2.20)	2.77 (1.60)	4.35 (2.20)	2.80 (1.62)
	Posttest, mean (SD)	3.06 (2.45)	4.06 (2.04)	2.53 (1.51)	4.02 (2.04)	2.51 (1.52)	4.15 (2.18)	2.72 (1.57)	4.13 (2.19)	2.72 (1.59)
	*z*	–4.80	–2.11	–2.36	–2.03	–2.09	–2.30	–0.92	–2.36	–1.37
	*P*	<.001	.04	.02	.04	.04	.02	.36	.02	.17
	Cohen’s *d*	0.14	.08	.10	.09	.07	.09	—	.10	—
**Control**											
	n	69	50	50	—	—	—	—	—	—
	Pretest, mean (SD)	2.83 (2.82)	4.19 (2.51)	2.58 (1.77)	—	—	—	—	—	—
	Posttest, mean (SD)	2.93 (2.58)	4.04 (2.54)	2.62 (1.71)	—	—	—	—	—	—
	*z*	–0.35	–0.73	–0.33	—	—	—	—	—	—
	*P*	.73	.47	.74	—	—	—	—	—	—
	Cohen’s *d*	—	—	—	—	—	—	—	—	—
**Paired Facebookers**											
	n	69	50	50	—	—	—	—	—	—
	Pretest, mean (SD)	1.86 (2.25)	4.27 (2.03)	2.9 (1.47)	—	—	—	—	—	—
	Posttest, mean (SD)	1.28 (1.81)	4.32 (1.73)	2.58 (1.43)	—	—	—	—	—	—
	z	–2.86	–.50	–2.38	—	—	—	—	—	—
	*P*	.004	.62	.018	—	—	—	—	—	—
	Cohen’s *d*	.28	NS	.22	—	—	—	—	—	—
**Control vs Paired Facebookers**											
	z	–2.24	.47	–1.9	—	—	—	—	—	—
	*P*	.025	.64	.057	—	—	—	—	—	—
	Cohen’s *d*	–0.34	NS	–0.38	—	—	—	—	—	—

**Table 2:** Cronbach alphas for the 4 questions on alcohol and its association with festive moments for pre- and posttests and for participant group in the second and third periods.

Type of participants	Period 2, Cronbach α		Period 3, Cronbach α	
	Pretest	Posttest	Pretest	Posttest
All Facebookers	.804	.754	.814	.822
Control	.882	.890	—	—
Paired Facebookers	.772	.708	—	—

### Effects on the Number of Glasses Consumed at Festive Moments

For the All Facebookers group, we observed a significant reduction in the number of glasses consumed at festive moments in the second period (*z*=–2.36, *P*=.02), but not the third (*z*=–0.92, *P*=.36).

For the control group, during the second period, we observed that there was no difference between the number of glasses at the beginning and at the end of the program (*z*=–0.33, *P*=.74). Concerning the Paired Facebookers*,* we observed a reduction between the beginning and the end of the period for the number of glasses consumed at festive moments (*z*=–2.38*, P*=.02). Concerning only the beginning of the period, we observed no difference between our control*s* and the Paired Facebookers in the number of glasses at festive moments (*z*=–0.79, *P*=.43).

Finally, when we compared the Paired Facebookers and the control group concerning the difference between the beginning and the end of period 2, we observed a difference in the number of glasses per festive moment indicating that the Paired Facebookers had a reduction in the declared number of glasses per festive moment compared to the control participants, but this did not reach statistical significance (*z*=–1.90, *P*=.06).

### Effect of the Prevention Program on the Reduction of the Association Between Alcohol Consumption and Festive Moments

For the third period, all participants could recall a mean 4.05 (SD 1.65) SMS text messages, made a mean 0.12 (SD 0.50) likes, and the number of days since registration was a mean 49.48 (SD 7.31) days. The linear regression analyses for the third period (*R* ^*2*^=–.008; *F*_4,303_=0.366, *P*=.83) did not show any effect of our variables of interest on the reduction of the association between alcohol and festive moments. Viewing the low number of participants with likes on the Facebook page in our population, we decided to explore only participants who had at least one participation on the Facebook page, for example, one like or a comment (n=22; likes: mean 1.68, SD 0.89). The linear regression analysis on this population (*R* ^*2*^=.253; *F*_4,21_=2.779, *P*=.06) (see Table 3) showed that only the number of days since registration in the program had a significant impact (*t*_21_=3.186, *P*=.005) on the difference concerning the association between festive moments and alcohol consumption.

**Table 3:** Linear regression analyses predicting reduction in association between alcohol and festive moments for participants who have participated at least once, controlling for age, number of Facebook likes, number of SMS text message recalls, and number of days since registration.

Predictors of reduction in association of alcohol and festive moments	β standardized	*t*_21_	*P*
Age	.072	0.354	.73
Number of likes	.043	0.207	.84
Number of correct SMS text message recalls	.019	0.086	.93
Number of days since registration	.624	3.186	.005

## Discussion

We examined the effectiveness of an SNS-based prevention program on social representations concerning alcohol consumption and party habits. This program was conducted through the Internet over 2 years with 651 participants during the first period, 301 during the second, and 305 during the last period. A total of 1011 different persons participated in this program and this evaluation. We had 69 students as a control group during the first period and 50 during the second period. The results showed a reduction in the link between alcohol and partying for our target population for the 3 periods. This result was not found for our control group. Interestingly, we observed that the declared number of glasses of alcohol consumed at festive moments diminished between the beginning and the end of the program for participants in the second period; however, this was not the case for our control group (or for our third period). Our results failed to show what factors influence this reduction for the population. But, when we explored only participants who had participated at least once, we showed that the reduction in the link between alcohol and festive moments is only influenced by the number of days since registration and not by the age, number of correct SMS text message recalls, or the number of likes on the page.

The results of this evaluation support the recent interest in Web-based programs for health policies [15] and their efficacy. They highlight the important role of this new way of communication for a medical approach. This type of support has many advantages for health promotion. First, our message could be spread to a large audience and not only population at school (pupils). This last type of population is intensively targeted by prevention programs because of the ease of access. The major interest of SNSs is that it could target other populations. There is no geographical limitation and rural environments could easily have access to this message. Another advantage is its accessibility at all times because it is the participant who can choose when they want to see the message. Moreover, we could adapt the message to a specific population.

Similar health programs via SNSs should use the specificity of this means of communication: the viral nature of information, the engagement of the participant to have an active action on messages, and all messages must be short (1-4 minutes for a video is recommended [25]).

Despite these interests, players in prevention must be very reactive to be effective because there are many changes in this domain and the habits of the young people of today will not remain constant for the long term. This was the case when young people left MSN Messenger (a social network platform) for Facebook during the early 2000s. For example, there is a new social network that is now used by young people: Twitter [26]. Communication on this social network is not exactly the same as on Facebook because users can only type very short messages (140 characters); thus, it is used differently.

There are some limitations to consider when interpreting the results of our study, which are essentially due to the ecological nature of this study. First, we could question our control population, who were mostly students and who may not be very similar to our sample. Their participation was completely uncompensated; thus, they did not have the same motivation as our experimental group. Another limitation is that we assessed the reduction in the association between alcohol and festive moments only for participants who successfully completed the 2 questionnaires. Despite our efforts, it must be noted that it is possible that this type of program will affect only participants who are sensitive to prevention messages. In all cases, we observed a positive impact on approximately 1100 persons.

Another limitation is that our program is not for a specific festive context. Indeed, Mallett et al [9] reviewed some experiences that showed that there are different high-risk events that are associated with alcohol consequences and probably with different consumption patterns. Futures studies should take these differences into account for a more efficient message.

Moreover, our measurements were based on self-reports. We cannot rule out that our results could be due to demand bias from the participants. Because most of our participants participated in a lottery, one cannot exclude the possibility that the participants consciously biased their responses to “please” the experimenter, particularly because there were high incentives for winning and alcohol consumption is especially sensitive to normative pressure [27]. However, Becona [28] found a close relationship between carbon monoxide levels and self-reported smoking rates in a smoking population. Moreover, Cherpitel [29] showed that self-reports of alcohol consumption measured in patients admitted to the emergency room were comparable to blood alcohol concentration. These 2 measures both predict behaviors in a similar manner, such as relating to alcohol-related violence [30; more details on Auvernight are available in 31]. Therefore, because confidentiality and anonymity are assured, we can be confident in our results.

Nevertheless, this ecological and powerful design allows us to believe that a long-term experiment to attest to the deep efficiency of this program could be implemented in the near future. Finally, this evaluation was focused only on alcohol, which was the major objective of this program of prevention, but we also used other messages of prevention (eg, cannabis, sexually transmitted infections). In future studies, it would be interesting to observe the impact of these messages on associated behavior and to observe the impact of the number and the quality of the messages on it. Our assessment failed to show what interaction modulates the association between festive moments and alcohol. Future studies need to explore this, probably utilizing a randomized design.

To conclude, our 2-year study exploring the efficacy of a prevention program on SNSs has shown encouraging results; we observed a reduction in the association of alcohol with festive moments and a reduction in the declared consumption of alcohol while partying. These results show that SNSs could be an interesting type of communication for promoting health and, more particularly, for impacting the social norms associated with alcohol consumption. In the future, the evaluation of the long-term impacts and the exploration of exactly which messages were the most efficient may be of interest.

## Abbreviations

SMS: short message service
SNS: social networking site
